# Characterization of Self-Assembled Virus-Like Particles of Merkel Cell Polyomavirus

**DOI:** 10.1371/journal.pone.0115646

**Published:** 2015-02-11

**Authors:** Tian-Cheng Li, Kenji Iwasaki, Harutaka Katano, Michiyo Kataoka, Noriyo Nagata, Kazumi Kobayashi, Tetsuya Mizutani, Naokazu Takeda, Takaji Wakita, Tetsuro Suzuki

**Affiliations:** 1 Department of Virology II, National Institute of Infectious Diseases, Tokyo, Japan; 2 Institute for Protein Research, Osaka University, Osaka, Japan; 3 Department of Pathology, National Institute of Infectious Diseases, Tokyo, Japan; 4 Cellular and Structural Physiology Institute, Nagoya University, Nagoya, Japan; 5 Research and Education center for Prevention of Global Infectious Diseases of Animals, Tokyo University of Agriculture and Technology, Tokyo, Japan; 6 Research Institute for Microbial Diseases, Osaka University, Osaka, Japan; 7 Department of Infectious Diseases, Hamamatsu University School of Medicine, Shizuoka, Japan; Kobe University, JAPAN

## Abstract

In our recombinant baculovirus system, VP1 protein of merkel cell polyomavirus (MCPyV), which is implicated as a causative agent in Merkel cell carcinoma, was self-assembled into MCPyV-like particles (MCPyV-LP) with two different sizes in insect cells, followed by being released into the culture medium. DNA molecules of 1.5- to 5-kb, which were derived from host insect cells, were packaged in large, ~50-nm spherical particles but not in small, ~25-nm particles. Structure reconstruction using cryo-electron microscopy showed that large MCPyV-LPs are composed of 72 pentameric capsomeres arranged in a T = 7 icosahedral surface lattice and are 48 nm in diameter. The MCPyV-LPs did not share antigenic determinants with BK- and JC viruses (BKPyV and JCPyV). The VLP-based enzyme immunoassay was applied to investigate age-specific prevalence of MCPyV infection in the general Japanese population aged 1–70 years. While seroprevalence of MCPyV increased with age in children and young individuals, its seropositivity in each age group was lower compared with BKPyV and JCPyV.

## Introduction

Merkel cell carcinoma is a rare but aggressive skin cancer of the elderly or immunosuppressed individuals. The recent identification of a novel human polyomavirus, Merkel cell polyomavirus (MCPyV), in Merkel cell carcinoma suggested implication of the virus in the pathogenesis of the disease. In many cases of Merkel cell carcinomas, MCPyV DNA was found to be clonally integrated in the genomes of the tumors. MCPyV DNA is known to be present in 70–80% of Merkel cell carcinomas in patients from different geographic locations [1–5].

Polyomaviruses are small, non-enveloped viruses that contain double-stranded, circular DNAs. The genome of MCPyV is 5.4 kb and encodes viral protein (VP) 1 and VP2 and a multiply-spliced T antigen oncogene locus [6]. Previous studies have shown that the surface of polyomaviruses such as SV40 and BK virus (BKPyV) are composed of only the major capsid protein VP1, and the viral particles are built of 72 capsomeres which are all pentamers of VP1 [7–9]. Although the molecular structure of MCPyV particles is expected to be analogous to those of other polyomaviruses, little is known about the structural features of MCPyV particles. Cell culture systems that support replication of MCPyV genome and production of infectious particles have been developed [10, 11]. However, the viral particles obtained from the replication systems have not been utilized for the structural analysis presumably because of their low yields. MCPyV is distantly related to the other human polyomaviruses; homologies of VP1 sequences between MCPyV and JC virus (JCPyV), BKPyV, and KI virus are 43, 45 and 26%, respectively. In fact, although it is known that antibodies against JCPyV, BKPyV and SV40 are frequently reactive each other, antibodies specific to MCPyV basically did not cross-react with other polyomaviruses [12].

In this study, we established an efficient production system of MCPyV-like particles (MCPyV-LP) by recombinant baculoviruses, in which MCPyV VP1 self-assembled into the MCPyV-LP, followed by being released into the culture medium. Structural features of purified MCPyV-LP were analyzed, and the MCPyV-LP was also applied to seroepidemiological investigation.

## Results and Discussion

### Self-assembly and release of MCPyV-LP in the culture medium of the baculovirus-insect cell system

**Fig 1:**
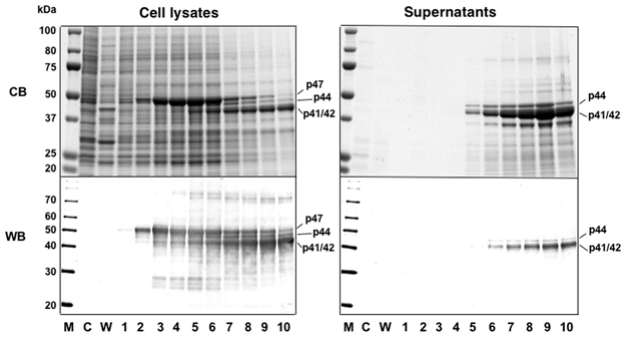
Time course of expression of MCPyV-VP1. Insect Tn5 cells were infected with AcMCPyV-VP1 and harvested for the indicated time periods. Five microliters of culture medium and samples of lysate from 10^5^ cells were analyzed by SDS-PAGE. Protein bands were visualized by Coomassie blue staining (CB; upper panels) and Western blotting with anti-MCPyV VP1 antiserum (WB; lower panel). M, molecular weight marker; C, mock-infected; W, wild-type baculovirus-infected cells; lanes 1 to 10, 1 to 10 days p.i.

While it has been shown that expression of MCPyV VP1 using the baculovirus expression system allows the formation of VLPs in insect cells and the VLPs are isolated from the cell lysates [13, 14], it can be advantageous for developing a simple procedure for purification of VLPs if the cells infected with the recombinant baculovirus are cultured with serum-free medium and VLPs produced are secreted into the culture supernatant. We thus explored the possibility of efficient production of MCPyV-LPs in insect *Trichoplusia ni*, BTL-Tn 5B1–4 (Tn5, also known as High Five) (Tn5) cells, which are known to possess high secretory capacity of recombinant proteins generated by baculoviruses, and their release into the serum-free culture medium [15]. Tn5 was infected with a recombinant baculovirus AcMCPyV-VP1 containing the entire MCPyV VP1 gene. The infected cells were harvested daily until 10 days post-infection (p.i.). Protein from infected cells and supernatant was analyzed by SDS—polyacrylamide gel electrophoresis (SDS—PAGE), followed by Coomassie blue staining and Western blotting. As shown in Fig. 1, a major protein with a molecular mass of 47 kDa (p47), identical to the entire MCPyV VP1, was detected in cells 2 days p.i. p47 declined over time as proteins of lower molecular weight were detected, such as 44- (p44) and 41/42- (p41/42) kDa (depicted in the top panel on the left in Fig. 1). p44 and p41/42, but not p47, were efficiently released into the culture supernatant (depicted in the top panel on the right in Fig. 1). These were not detectable in mock-infected or wild-type baculovirus-infected cells by Western blotting (as depicted in the lower panels of Fig. 1). Protein microsequencing analysis, which was carried out by Edman automated degradation of the purified proteins on an Applied Biosystems model 477 protein sequencer, demonstrated that the N-terminal amino acid sequence of p44 and p41/42 was Ala-Pro-Lys-Arg-Lys, identical to the first 5 residues of MCPyV VP1, suggesting that the multiple forms of MCPyV VP1 found within the cells and their supernatant were generated by deletion of its C-terminal ~50 residues.

**Fig 2:**
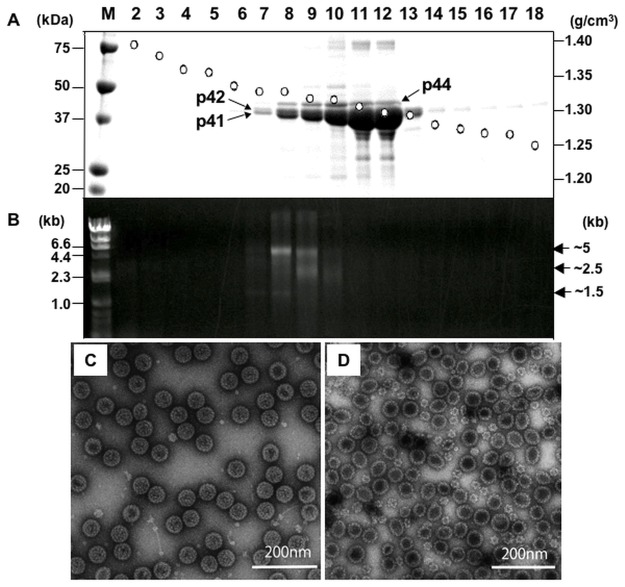
Purification and structural characterization of MCV-LP. The supernatant of AcMCPyV-VP1-infected cells was centrifuged for 3 h at 32,000 rpm in a Beckman SW32Ti rotor. The pellet was then re-suspended in 500 μl medium, and then purified by CsCl equilibrium density gradient centrifugation. Aliquots from each fraction were analyzed by SDS-PAGE and stained with Coomassie blue (A). Nucleic acids extracted from each fraction were analyzed by agarose gel electrophoresis (B). Fractions 7–9 (C) and 11–13 (D) were pooled, respectively, followed by staining with 2% uranyl acetate and observation by EM. Bars, 200 nm.

To examine whether p44 and p41/42 of MCPyV VP1 formed into VLPs, the supernatant of AcMCPyV-VP1-infected cells was harvested at 7 days p.i. and subjected to CsCl gradient centrifugation. p44 and p41/42 were detected in fractions of 1.29–1.32 g/cm^3^ (Fr. 7–13, Fig. 2A). Spherical particles of uniform size with a diameter of ~50 nm were observed in fractions of 1.31–1.32 g/cm^3^ (Fr. 7–10) by transmission electron microscopy (TEM) (Fig. 2C). In contrast, two kinds of VLPs with sizes at ~25 nm and ~50 nm were detectable in 1.29–1.30 g/cm^3^ fractions (Fr. 11–13)(Fig. 2D). It has been demonstrated that the VP1 assembly of polyomaviruses is calcium dependent [16–18] and that, while small T = 1 VLPs can be synthesized without calcium, the larger T = 7 particles require the calcium ion for their assembly [19, 20]. It may be thus likely that population ratio of large and small VLPs of MCPyV generated is affected by the calcium concentration in cells infected with the recombinant baculovirus. Together with the finding from N-terminal amino acid sequence analysis as described above, it appears that the C-terminal region of MCPyV VP1 has no influence on the capsid assembly.

Although preparation of MCPyV-LPs from cell lysates infected with recombinant baculoviruses has been reported [13, 14], to the best of our knowledge, this is the first study to demonstrate purification of MCPyV-LPs secreted into the serum-free culture medium. This has the advantage of being a simpler procedure by which to isolate and purify VLPs.

### Structural features of MCPyV-LPs

The N-terminal region of VP1 of polyomaviruses has a role in encapsidation of the viral genome. It has been shown that the viral VP1 VLPs produced in bacterial or insect cells contain endogenous nucleic acids, suggesting that DNA-VP1 interaction may have an influence on particle assembly of polyomaviruses [21–24]. To determine whether cellular nucleic acids were packaged into the MCPyV-LPs, nucleic acids were extracted from purified MCPyV-LPs, followed by agarose gel electrophoresis. As shown in Fig. 2B, nucleic acids of ~5 kb, 2.5 kb and ~1.5 kb were detected in fractions 7–10, but not in fractions 11–13. The nucleic acids detected were sensitive to DNase treatment. By applying a rapid genome sequencing technique based on cDNA representational difference analysis [25], we identified several host insect cell-derived DNA fragments as nucleic acids incorporated in the MCPyV-LPs (data not shown). Interestingly, the size and shape of VLPs appeared to vary according to whether or not DNA was packaged. It may be that DNA acts as an allosteric effector and that the interaction between DNA and VP1 supports the formation of ~50-nm particles.

**Fig 3:**
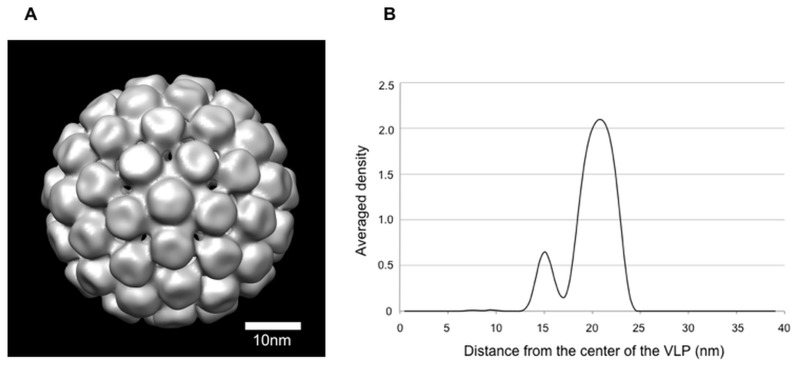
Cryo-EM. (A) A reconstructed 3D image of the MCPyV-LP. UCSF Chimera was used to visualize this structure [38]. The threshold for isosurface representation was calculated such that the volume could include 100% of the mass of the MCPyV-LP. Bar, 10 nm. (B) One-dimensional profile demonstrating the rotationally averaged density of the reconstructed volume. Density is plotted against distance from the center of the virus particle. Values outside the particle dimensions were masked out.

To further study the structure of MCPyV-LPs, we visualized the three-dimensional (3D) structure of ~50-nm VLPs, whose size is close to native polyomaviruses, using cryo-electron microscopy (cryo-EM). The 3D reconstruction of the MCPyV-LPs appeared similar to those of the T = 7 VLPs of BKPyV [9] as well as native polyomaviruses [26]. The projected density map clearly showed that MCPyV-LPs are composed of 72 capsomeres and the pentameric capsomeres occupy both pentavalent and hexavalent positions arranged in a right-handed T = 7 icosahendral lattices (Fig 3A).

A high resolution structure of MCPyV VP1 unassembled pentamer is available online (Protein Data Bank, code 4FMG) [27]. We confirmed that a pseudoatomic model for the capsid structure generated from this pentamer structure fitted well with our 3D structure of MCPyV-LP (data not shown). Somewhat unexpectedly, the maximum diameter of each MCPyV-LP was estimated at 48 nm based on a rotationally averaged density calculated using SPIDER system (Fig. 3B). The size of MCPyV-LP seems slightly smaller than those of BKPyV (50.6 nm), SV40 (49.4 nm) and murine polyomavirus (49.5 nm) [9, 26]. In addition to the major peak, a minor density peak was found at approximately 15 nm distance from the center of the VLP. Considering our evidence that cell-derived DNAs were contained in the MCPyV-LPs (Fig 2B) as well as structural information for other polyomaviruses [23], this density peak might express DNA population inside the particles.

### Application of VLPs to seroepidemiology

**Fig 4:**
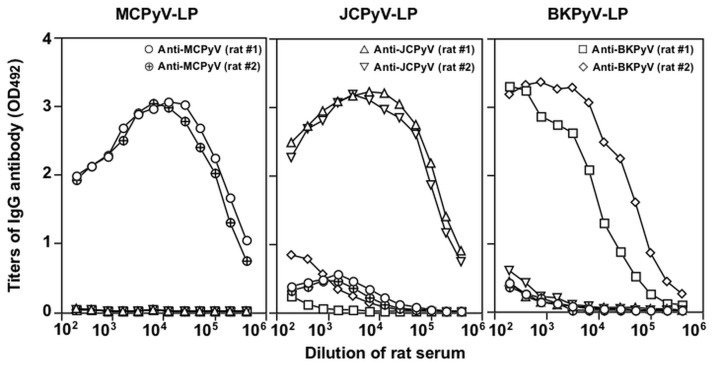
Antigenic cross-reactivity among MCPyV-, BKPyV- and JCPyV-LPs. Hyperimmune sera were obtained from rats that were immunized with MCPyV-, BKPyV- and JCPyV-LPs, respectively. Titers of anti-MCPyV-, BKPyV- and JCPyV-LP-IgG were determined by ELISA.

In several studies, VLPs of polyomaviruses including MCPyV were applied to detection of the viral antibodies in the general population of the United States and Europe [13, 14, 28–32]. However, little is known about seroepidemiology of MCPyV in Asian countries to date, except for one report from China [33]. First, to explore the antigenic cross-reactivity among BKPyV, JCPyV and MCPyV, rats were immunized with BKPyV-LPs, JCPyV-LPs or MCPyV-LPs. After three injections without adjuvant use, all of the rats elicited a high level of IgG antibodies against each homologus antigen, and IgG titers by antibody enzyme-linked immunosorbent assay (ELISA) reached as high as 1:409,600 (BKPyV), 1: 1,819,200 (JCPyV) and 1:1,819,200 (MCPyV), respectively (Fig. 4). Anti-MCPyV-LP antiserum did not react with BKPyV-LP or JCPyV-LP. Only low titers of cross-reactive antibodies against MCPyV-LP were detected in anti-BKPyV-LP and anti-JCPyV-LP antisera. These results suggest that MCPyV does not share significant antigenic determinants with BKPyV and JCPyV. The VP1 amino acid sequence similarity of MCPyV to those of BKPyV and JCPyV used in this study were 45% and 43%, respectively. Further detailed analyses of 3D reconstruction of VLPs and VP1 peudoatomic models may help us in addressing question regarding location of amino acid(s) to determine the difference in antigenic reactivity among MCPyV, BKPyV and JCPyV.

Next, VLP-based ELISA was applied to investigate age-specific prevalence of infections with MCPyV, BKPyV and JCPyV in Japan. Seropositivity of IgG antibodies against these polyomaviruses in the general Japanese population aged 1–70 years (529 males, 521 females) was determined (Table 1). Overall, anti-MCPyV antibodies were detected in 43% of subjects, which was lower than the observed prevalence of anti-BKPyV (68%) and anti-JCPyV (51%) antibodies. The age-related seroprevalence of MCPyV antibodies was assessed and found to be 13%, 23%, 38% and 43%, respectively, for children and young individuals aged 1–5, 6–10, 11–15 and 16–20 years old. Gender-specific differences were not evident. Compared to studies reported from research groups in the United States, Europe and China [13, 14, 28–33], seropositivity for anti-MCPyV antibodies is less frequent among children and adults in our study at any given age. Although it cannot be ruled out the possibility that the lower seroprevalence could be due to lower assay sensitivity, geographical factors might explain the observed differences in results. No children aged 1 (n = 8) or 2 (n = 7) years were positive for anti-MCPyV antibodies, suggesting that vertical transmission of MCPyV occurs rarely if at all. Alternatively, the lack of responsiveness could be explained by the idea that infants are infected but have not yet mounted antibody responses against the virus. Given high seropositivity for anti-BKPyV antibody at age 1–10, it is possible that the transmission route of MCPyV is different from that of BKPyV.

**Table 1:** Seropositivity of IgG antibodies against MCPyV, BKPyV and JCPyV in the general Japanese population aged 1–70 years.

	MCPyV					BKPyV					JCPyV				
Ages	Total	Male		Female		Total	Male		Female		Total	Male		Female	
	%	%	(pos/No)*	%	(pos/No)	%	%	(pos/No)	%	(pos/No)	%	%	(pos/No)	%	(pos/No)
1~5	12	11	(4/38)	14	(5/36)	46	45	(17/38)	47	(17/36)	20	24	(9/38)	17	(6/36)
6~10	23	18	(7/39)	28	(11/39)	82	79	(31/39)	85	(33/39)	19	23	(9/39)	15	(6/39)
11~15	37	43	(16/37)	32	(12/37)	86	81	(30/37)	92	(34/37)	32	30	(11/37)	35	(13/37)
16~20	43	42	(16/38)	45	(17/38)	71	68	(26/38)	74	(28/38)	43	42	(16/38)	45	(17/38)
21~25	37	46	(12/26)	29	(10/34)	75	69	(18/26)	79	(27/34)	38	35	(9/26)	41	(14/34)
26~30	55	50	(17/34)	62	(16/26)	72	74	(25/34)	69	(18/26)	37	41	(14/34)	31	(8/26)
31~35	34	33	(13/40)	35	(14/40)	74	75	(30/40)	73	(29/40)	56	53	(21/40)	60	(24/40)
36~40	45	50	(10/20)	40	(8/20)	60	65	(13/20)	55	(11/20)	55	50	(10/20)	60	(12/20)
41~45	44	43	(13/30)	46	(11/24)	63	47	(14/30)	83	(20/24)	59	63	(19/30)	54	(13/24)
46~50	55	43	(13/30)	64	(23/36)	62	47	(14/30)	75	(27/36)	64	70	(21/30)	58	(21/36)
51~55	43	50	(15/30)	36	(12/33)	59	60	(18/30)	58	(19/33)	57	53	(16/30)	61	(20/33)
56~60	57	57	(26/46)	57	(24/42)	70	67	(31/46)	74	(31/42)	67	67	(31/46)	67	(28/42)
61~65	47	49	(30/61)	45	(25/55)	68	59	(36/61)	78	(43/55)	67	69	(42/61)	65	(36/55)
66~70	57	55	(33/60)	59	(36/61)	64	63	(38/60)	66	(40/61)	74	78	(47/60)	69	(42/61)
Total	43	43	(225/529)	43	(224/521)	68	64	(341/529)	72	(377/521)	51	52	(275/529)	50	(260/521)

* numbers of seropositive samples/numbers of samples tested

In summary, we demonstrated simple and efficient purification of MCPyV-LP expressed by recombinant baculovirus, from serum-free cultured medium. Cryo-EM analysis identified a major population of MCPyV-LP that were 48 nm in diameter and consisted of T = 7 icosahedral particles. MCPyV is still difficult to grow in cultured cells and the application of recombinant viral proteins is prerequisite for studies of particle structure, diagnosis and vaccine development. Given the high immunoreactivity but low antigenic cross-reactivity, the VLP-based ELISA would be useful for serological diagnosis of MCPyV infection. The MCPyV-LP may also be a potential candidate for vaccine.

## Materials and Methods

### Construction of a recombinant baculovirus and expression of MCPyV VP1 protein

MCPyV gene (GenBank accession no. FJ464337) was isolated from a case of MCPyV-positive Kaposi’s sarcoma [34], and the entire VP1 gene of the MCPyV genome covering nucleotides 3156 to 4427 was amplified by PCR with forward primer MCV-D1 (5’-GCGGATCCATGGCACCCAAAAGAAAAG-3’), which contains the *Bam*HI site before the start codon, and reverse primer MCV-U1 (5’-GGTCTAGATCATAATTCTTGTGTTTG-3’), which contains the *Xba*I site after the stop codon. The MCPyV VP1 fragment was first digested with *Bam*HI and then partially digested with *Xba*I. A transfer plasmid pVL1393/MCPyV-VP1 was constructed by inserting the purified MCPyV VP1 fragment into pVL1393 (Pharmingen).

Insect cells *Spodoptera frugiperda* (Sf9)(Riken Cell Bank, Tsukuba, Japan) were cotransfected with the linearized wild-type *Autographa californica* nuclear polyhedrosis virus DNA (BaculoGold 21100D, Pharmingen) and pVL1393-MCPyV-VP1 by the lipofectin-mediated method as specified by the manufacturer (GIBCO-BRL). The cells were incubated at 26.5°C in TC-100 medium (GIBCO-BRL) supplemented with 8% fetal bovine serum and 0.26% bactotryptose phosphate broth (Difco Laboratories). The recombinant virus was plaque-purified three times in Sf9 cells and designated as AcMCPyV-VP1. To achieve large-scale expression, Tn5 cells (Invitrogen) were infected with the recombinant baculovirus Ac MCPyV-VP1 at a multiplicity of infection of 10 and cultured in EX-CELL 405 medium (JRH Biosciences) at 26.5°C.

### Western blot analysis

The proteins in the cell lysates and culture media were separated by 12.5% SDS—PAGE and were electrophoretically transferred onto a nitrocellulose membrane. The membrane was then blocked with 5% skim milk in 50 mM Tris—HCl (pH 7.4), 150 mM NaCl, and reacted with a rabbit anti-MCPyV VP1 polyclonal antibody. Detection of rabbit IgG antibody was achieved by using alkaline phosphatase-conjugated goat anti-rabbit immunoglobulin (Chemicon International). Nitroblue tetrazolium chloride and 5-bromo-4-chloro-3-indolyl phosphate P-toluidine were used as coloring agents (Bio-Rad Laboratories). The MCPyV VP1 antigen to generate a rabbit anti-MCPyV VP1 polyclonal antibody was prepared as follows. A glutathione S transferase-MCPyV VP1 fusion protein was bacterially produced from pGEX5X/MCPyVVP1, where MCPyV VP1 gene was inserted into the *Bam*HI-*Xho*I site of pGEX5X-2 (GE Healthcare), and purified by glutathione Sepharose chromatography.

### Purification of VLPs

AcMCPyV-VP1-infected Tn5 cells were harvested on day 7 p.i.. The cells, their debris, and the progeny baculoviruses were removed by centrifugation at 10,000 x *g* for 60 min. The supernatant was then spun at 32,000 rpm for 3 h in a Beckman SW32 Ti rotor, and the resulting pellet was resuspended in 4.5 ml EX-CELL 405 at 4°C overnight. After mixing with 2.1 g of CsCl, the sample was centrifuged at 35,000 rpm for 24 h at 10°C in a Beckman SW55 rotor. The gradient was fractionated into 250 μl aliquots, and each fraction was weighed in order to estimate the buoyant density and isopycnic point. Each fraction was diluted with Ex-cell 405 and centrifuged for 2 h at 50,000 rpm in a Beckman TLA55 rotor to sediment MCPyV-LP. The pellet was dissolved in EX-CELL 405 medium, mixed with 2.1 g CsCl in a final volume of 5 ml, and used for CsCl gradient centrifugation.

### N-terminal amino acid sequence analysis

Purified VLPs were subjected to the N-terminal aa microsequencing, which was carried out using 100 pmol of protein by Edman automated degradation on an Applied Biosystems model 477 protein sequencer.

### Electron microscopy

For TEM analysis, purified MCPyV-LPs were placed on a carbon-coated grid for 45 seconds, rinsed with distilled water, stained with a 2% uranyl acetate solution and examined with a JEOL TEM-1400 electron microscope operating at 80 kV. For cryo-EM analysis, purified MCPyV-LPs were plunge frozen on Quantifoil holey grids (Quantifoil Micro Tools GmbH) that were glow-discharged in advance. Images of ice-embedded specimen were collected at liquid-helium temperature on a JEM-Z2100FC cryo-electron microscope operated at 200 kV. The pixel size of cryo-EM images recorded with a TVIPS (Tietz Video and Image Processing Systems GmbH) 8K × 8K CMOS camera was calibrated to be 0.13 nm/pixel using Tobacco Mosaic Virus as a standard.

### Nucleic acid extraction from MCPyV-LPs

Nucleic acids in the purified MCPyV-LPs were prepared using a MagNA Pure LC Total Nucleic Acid Isolation kit (Roche Diagnostics). Fifty μl of the extract was treated with DNase I (final concentration; 0.01 mg/ml) (Sigma-Aldrich) at 37°C for 30 min or with RNase A (final concentration; 0.5 μg/ml) at 37°C for 1 h, followed by analyzing on 1% agarose gel electrophoresis.

### Image Processing

The single-particle reconstruction was performed using EMAN [35]. 342 picked up particles were binned by a factor of four using *shrink* option of *proc2d*, which is one of EMAN commands. The initial model produced by *starticos* command was refined by 150 times using *refine* with the combination of low-pass filter. The resolution of the map that was assessed by the 0.5 Fourier Shell Correlation criterion indicated 4nm. The average density was calculated using the single-particle software system (SPIDER) [36]. Firstly, threshold density was calculated to incorporate the expected 16,765 kDa mass of the MCPyV. Secondary, the extra density was mask out. Thirdly, the average density was calculated by *RO* of SPIDER command.

### Human serum samples

Specimens tested for MCPyV serology consisted of 1050 serum samples collected from healthy Japanese individuals, which were supplied from Serum Bank of National Institute of Infectious Diseases, Japan. The age ranged from 1 to 70 years old and 50.4% (529/1050) was male, and 49.6% (521/1050) was female.

### Hyperimmune sera against MCPyV-, BKPyV- or JCPyV-LP

The animal procedures were approved by the Committee on Biosafety and Animal Handling Regulations of the National Institute of Infectious Diseases. Rats (Wistar, 12 weeks old, female) were immunized with the purified MCPyV-, BKPyV- or JCPyV-LP by intramuscular injection with a dose of 100 μg VLP per rat. Booster and last injections with the same dose were done after 4 and 6 weeks, respectively. All the injections were carried out without any adjuvant. The animals were bled 1 week after the booster injection.

### Detection of anti- MCPyV, BKPyV and JCPyV IgG antibody by ELISA

Flat-bottom 96-well polystyrene microplates (Immulon 2; Dynex Technologies) were coated with the purified MCPyV-LPs, BKPyV-LPs and JCPyV-LPs (1 μg/ml, 100 μl/well) and incubated overnight at 4°C. Unbound VLPs were removed, and the plates were washed twice with 10 mM phosphate-buffered saline containing 0.05% Tween 20 (PBS-T) and then blocked with 200 μl of 5% skim milk (Difco Laboratories) dissolved in PBS-T for 1 h at 37°C. After washing four times with PBS-T, diluted pig, rabbit, or guinea pig (100 μl/well) serum samples were added in duplicate. The plates were incubated at 37°C for 1 h and washed three times as described above. The wells were incubated with 100 μl of horseradish peroxidase-conjugated goat anti-human IgG (H+L) (KPL) (1:5,000 dilution), or horseradish peroxidase-conjugated goat anti-rabbit IgG (Cappel) (1:2,000 dilution), diluted with PBS-T containing 1% skim milk. The plates were incubated at 37°C for 1 h and washed four times with PBS-T. The substrate orthophenylenediamine (100 μl) (Sigma-Aldrich) and H_2_O_2_ were added to each well. The plates were incubated in a dark room at room temperature for 30 min, then 50 μl of 4N H_2_SO_4_ was added into each well. Absorbance was measured at 492 nm. The cut-off value for seropositivity was defined as optical density values greater than the mean plus 3 standard deviations of the reactivity of serum samples from populations negative in Western blotting (50 samples tested for MCPyV, BKPyV and JCPyV, respectively), as described previously [37]. OD value of negative IgG antibodies to MCPvV, BKPyV and JCPyV ranged from 0.008 to 0.264 (mean ± SD = 0.084 ± 0.038), 0.012 to 0.299 (mean ± SD = 0.076 ± 0.043), 0.016 to 0.291 (mean ± SD = 0.082 ± 0.040), respectively. Therefore, test samples were considered positive when the absorbance was ≥0.200.

## References

[1] DeCaprio JA, Garcea RL (2013) A cornucopia of human polyomaviruses. Nat Rev Microbiol 11: 264–276. 10.1038/nrmicro2992 23474680 PMC3928796

[2] Spurgeon ME, Lambert PF (2013) Merkel cell polyomavirus: a newly discovered human virus with oncogenic potential. Virology 435: 118–130. 10.1016/j.virol.2012.09.029 23217622 PMC3522868

[3] Gjoerup O, Chang Y (2010) Update on human polyomaviruses and cancer. Adv Cancer Res 106: 1–51. 10.1016/S0065-230X(10)06001-X 20399955

[4] Becker JC, Houben R, Ugurel S, Trefzer U, Pfohler C, et al (2009) MC polyomavirus is frequently present in Merkel cell carcinoma of European patients. J Invest Dermatol 129: 248–250. 10.1038/jid.2008.198 18633441

[5] Kassem A, Schopflin A, Diaz C, Weyers W, Stickeler E, et al (2008) Frequent detection of Merkel cell polyomavirus in human Merkel cell carcinomas and identification of a unique deletion in the VP1 gene. Cancer Res 68: 5009–5013. 10.1158/0008-5472.CAN-08-0949 18593898

[6] Feng H, Shuda M, Chang Y, Moore PS (2008) Clonal integration of a polyomavirus in human Merkel cell carcinoma. Science 319: 1096–1100. 10.1126/science.1152586 18202256 PMC2740911

[7] Stehle T, Gamblin SJ, Yan Y, Harrison SC (1996) The structure of simian virus 40 refined at 3.1 A resolution. Structure 4: 165–182. 8805523 10.1016/s0969-2126(96)00020-2

[8] Liddington RC, Yan Y, Moulai J, Sahli R, Benjamin TL, et al (1991) Structure of simian virus 40 at 3.8-A resolution. Nature 354: 278–284. 10.1038/354278a0 1659663

[9] Li TC, Takeda N, Kato K, Nilsson J, Xing L, et al (2003) Characterization of self-assembled virus-like particles of human polyomavirus BK generated by recombinant baculoviruses. Virology 311: 115–124. 12832209 10.1016/s0042-6822(03)00141-7

[10] Neumann F., Borchert S., Schmidt C., Reimer R., Hohenberg H., Fischer N., and Grundhoff A.. 2011 Replication, gene expression and particle production by a consensus Merkel Cell Polyomavirus (MCPyV) genome. PLOS ONE 6:e29112 10.1371/journal.pone.0029112 22216177 PMC3246459

[11] Schowalter R. M., Pastrana D. V., and Buck C. B.. 2011 Glycosaminoglycans and sialylated glycans sequentially facilitate Merkel cell polyomavirus infectious entry. PLOS Pathog 7:e1002161 10.1371/journal.ppat.1002161 21829355 PMC3145800

[12] Tolstov YL, Pastrana DV, Feng H, Becker JC, Jenkins FJ, et al (2009) Human Merkel cell polyomavirus infection II. MCV is a common human infection that can be detected by conformational capsid epitope immunoassays. Int J Cancer 125: 1250–1256. 10.1002/ijc.24509 19499548 PMC2747737

[13] Touze A, Gaitan J, Arnold F, Cazal R, Fleury MJ, et al (2010) Generation of Merkel cell polyomavirus (MCV)-like particles and their application to detection of MCV antibodies. J Clin Microbiol 48: 1767–1770. 10.1128/JCM.01691-09 20181914 PMC2863896

[14] Viscidi RP, Rollison DE, Sondak VK, Silver B, Messina JL, et al (2011) Age-specific seroprevalence of Merkel cell polyomavirus, BK virus, and JC virus. Clin Vaccine Immunol 18: 1737–1743. 10.1128/CVI.05175-11 21880855 PMC3187023

[15] Li TC, Yamakawa Y, Suzuki K, Tatsumi M, Razak MA, et al (1997) Expression and self-assembly of empty virus-like particles of hepatitis E virus. J Virol 71: 7207–7213. 9311793 10.1128/jvi.71.10.7207-7213.1997 PMC192060

[16] Salunke DM, Caspar DL, Garcea RL (1986) Self-assembly of purified polyomavirus capsid protein VP1. Cell 46: 895–904. 3019556 10.1016/0092-8674(86)90071-1

[17] Salunke DM, Caspar DL, Garcea RL (1989) Polymorphism in the assembly of polyomavirus capsid protein VP1. Biophys J 56: 887–900. 10.1016/S0006-3495(89)82735-3 2557933 PMC1280588

[18] Schmidt U, Rudolph R, Bohm G (2000) Mechanism of assembly of recombinant murine polyomavirus-like particles. J Virol 74: 1658–1662. 10644335 10.1128/jvi.74.4.1658-1662.2000 PMC111640

[19] Nilsson J, Miyazaki N, Xing L, Wu B, Hammar L, et al (2005) Structure and assembly of a T = 1 virus-like particle in BK polyomavirus. J Virol 79: 5337–5345. 10.1128/JVI.79.9.5337-5345.2005 15827148 PMC1082729

[20] Kanesashi SN, Ishizu K, Kawano MA, Han SI, Tomita S, et al (2003) Simian virus 40 VP1 capsid protein forms polymorphic assemblies in vitro. J Gen Virol 84: 1899–1905. 12810885 10.1099/vir.0.19067-0

[21] Li PP, Nakanishi A, Shum D, Sun PC, Salazar AM, et al (2001) Simian virus 40 Vp1 DNA-binding domain is functionally separable from the overlapping nuclear localization signal and is required for effective virion formation and full viability. J Virol 75: 7321–7329. 10.1128/JVI.75.16.7321-7329.2001 11462004 PMC114967

[22] Soussi T (1986) DNA-binding properties of the major structural protein of simian virus 40. J Virol 59: 740–742. 3016336 10.1128/jvi.59.3.740-742.1986 PMC253252

[23] Gillock ET, Rottinghaus S, Chang D, Cai X, Smiley SA, et al (1997) Polyomavirus major capsid protein VP1 is capable of packaging cellular DNA when expressed in the baculovirus system. J Virol 71: 2857–2865. 9060642 10.1128/jvi.71.4.2857-2865.1997 PMC191411

[24] Pawlita M, Muller M, Oppenlander M, Zentgraf H, Herrmann M (1996) DNA encapsidation by viruslike particles assembled in insect cells from the major capsid protein VP1 of B-lymphotropic papovavirus. J Virol 70: 7517–7526. 8892870 10.1128/jvi.70.11.7517-7526.1996 PMC190819

[25] Mizutani T, Endoh D, Okamoto M, Shirato K, Shimizu H, et al (2007) Rapid genome sequencing of RNA viruses. Emerg Infect Dis 13: 322–324. 10.3201/eid1302.061032 17479903 PMC2725858

[26] Rayment I, Baker TS, Caspar DL, Murakami WT (1982) Polyoma virus capsid structure at 22.5 A resolution. Nature 295: 110–115. 6276752 10.1038/295110a0 PMC4144041

[27] Neu UH, Hengel BS, Blaum RM, Schowalter D, Macejak M, et al (2012) Structures of Merkel cell polyomavirus VP1 complexes define a sialic acid binding site required for infection. PLOS Pathog 8: e1002738 10.1371/journal.ppat.1002738 22910713 PMC3406085

[28] Nicol JT, Robinot R, Carpentier A, Carandina G, Mazzoni E, et al (2013) Age-specific seroprevalences of merkel cell polyomavirus, human polyomaviruses 6, 7, and 9, and trichodysplasia spinulosa-associated polyomavirus. Clin Vaccine Immunol 20: 363–368. 10.1128/CVI.00438-12 23302741 PMC3592346

[29] Kean JM, Rao S, Wang M, Garcea RL (2009) Seroepidemiology of human polyomaviruses. PLOS Pathog 5: e1000363 10.1371/journal.ppat.1000363 19325891 PMC2655709

[30] Pastrana DV, Tolstov YL, Becker JC, Moore PS, Chang Y, et al (2009) Quantitation of human seroresponsiveness to Merkel cell polyomavirus. PLOS Pathog 5: e1000578 10.1371/journal.ppat.1000578 19750217 PMC2734180

[31] Faust H, Pastrana DV, Buck CB, Dillner J, Ekstrom J (2011) Antibodies to Merkel cell polyomavirus correlate to presence of viral DNA in the skin. J Infect Dis 203: 1096–1100. 10.1093/infdis/jiq173 21450999

[32] Carter JJ, Paulson KG, Wipf GC, Miranda D, Madeleine MM, et al (2009) Association of Merkel cell polyomavirus-specific antibodies with Merkel cell carcinoma. J Natl Cancer Inst 101: 1510–1522. 10.1093/jnci/djp332 19776382 PMC2773184

[33] Zhang CF, Liu Z, He Q, Deng Y, Pan Y, et al (2014) Seroprevalence of Merkel cell polyomavirus in the general rural population of Anyang, China. PLOS ONE 9: e106430 10.1371/journal.pone.0106430 25184447 PMC4153645

[34] Katano H, Ito H, Suzuki Y, Nakamura T, Sato Y, et al (2009) Detection of Merkel cell polyomavirus in Merkel cell carcinoma and Kaposi’s sarcoma. J Med Virol 81: 1951–1958. 10.1002/jmv.21608 19774683

[35] Ludtke SJ (2010) 3-D structures of macromolecules using single-particle analysis in EMAN. Methods Mol Biol 673: 157–173. 10.1007/978-1-60761-842-3_9 20835797

[36] Shaikh TR, Gao H, Baxter WT, Asturias FJ, Boisset N, et al (2008) SPIDER image processing for single-particle reconstruction of biological macromolecules from electron micrographs. Nat Protoc 3: 1941–1974. 10.1038/nprot.2008.156 19180078 PMC2737740

[37] Matsuura Y, Suzuki M, Yoshimatsu K, Arikawa J, Takashima I, et al (2007) Prevalence of antibody to hepatitis E virus among wild sika deer, Cervus nippon, in Japan. Arch Virol 152: 1375–81. 17431737 10.1007/s00705-007-0965-6

[38] Pettersen EF, Goddard TD, Huang CC, Couch GS, Greenblatt DM, et al (2004) UCSF Chimera—a visualization system for exploratory research and analysis. J Comput Chem 25: 1605–1612. 10.1002/jcc.20084 15264254

